# Age-related immune alterations and cerebrovascular inflammation

**DOI:** 10.1038/s41380-021-01361-1

**Published:** 2021-10-28

**Authors:** Carson E. Finger, Ines Moreno-Gonzalez, Antonia Gutierrez, Jose Felix Moruno-Manchon, Louise D. McCullough

**Affiliations:** 1 Department of Neurology, McGovern Medical School, UTHealth Science Center at Houston, Houston, TX USA; 2 Department of Cell Biology, Genetics and Physiology, Instituto de Investigacion Biomedica de Malaga-IBIMA, Faculty of Sciences, Malaga University, Malaga, Spain; 3 Biomedical Research Networking Center on Neurodegenerative Diseases (CIBERNED), Malaga, Spain

**Keywords:** Diseases, Neuroscience

## Abstract

Aging is associated with chronic systemic inflammation, which contributes to the development of many age-related diseases, including vascular disease. The world’s population is aging, leading to an increasing prevalence of both stroke and vascular dementia. The inflammatory response to ischemic stroke is critical to both stroke pathophysiology and recovery. Age is a predictor of poor outcomes after stroke. The immune response to stroke is altered in aged individuals, which contributes to the disparate outcomes between young and aged patients. In this review, we describe the current knowledge of the effects of aging on the immune system and the cerebral vasculature and how these changes alter the immune response to stroke and vascular dementia in animal and human studies. Potential implications of these age-related immune alterations on chronic inflammation in vascular disease outcome are highlighted.

## Introduction

Aging is an inevitable biological process that affects all organs and cells, including the immune system, the cerebral vasculature, and the brain. Aging results in significant and complex changes to both the innate and adaptive immune system [1]. With aging, there is a decline in immune system efficacy, and this immunosenescence results in greater susceptibility to infections [2]. The lifetime risk of cerebrovascular disease and vascular dementia has increased, driven by the increasing age of the global population [3]. Age is a risk factor for stroke-related complications such as infections, cardiac events, and delirium, as well as mortality [4]. Elderly patients also have higher rates of post-stroke depression and cognitive decline [5]. Stroke accelerates the progression of neurodegenerative diseases, such as Alzheimer’s disease (AD), and is a significant contributor to vascular dementia [6, 7]. The immune system is a key player in both the acute and chronic response to stroke, and age-related alterations in the immune system contribute to the poorer outcomes seen in older patients [8]. The development of novel strategies to target or reverse this detrimental immune response is an active area of investigation.

This review summarizes recent studies examining immune and vascular senescence and how these age-related changes can affect the immune response to stroke and other cerebrovascular diseases. A better understanding of age-related immune alterations in vascular disease will create a foundation for developing therapies applicable to the majority of patients affected by these diseases, the elderly.

## Brain immunosenescence

Senescence is an irreversible replicative-arrest state of cells, leading to changes in gene expression and phenotype that alter the function of neighboring cells [9]. Senescent cells release pro-inflammatory signals (e.g., Interleukins (IL), IL-1α, IL-1β, IL-6, and IL-8) that are referred to as the “senescence-associated secretory phenotype” (SASP) [10]. Accumulation of senescent cells during aging promotes chronic inflammation and tissue dysfunction and is an essential contributor to the progression of age-associated diseases (i.e., AD and atherosclerosis). Endothelial, epithelial, and stromal cells can express the SASP [11], leading to the recruitment of immune cells and an increased pro-inflammatory milieu [12]. Glial cells also undergo senescence both in vitro and in vivo, contributing to age-related neuroinflammation and vascular dysfunction. Enhancing the clearance of senescent microglia and astrocytes using genetic or pharmacological approaches reduced tau aggregation and led to the preservation of cognitive function in murine models [13]. This suggests that there is potential to reverse some of the detrimental immune responses seen with aging.

Franceschi et al. [14] put forth a theory that aging results in a chronic increase in systemic inflammation, termed “inflammaging.” Chronic inflammation caused by aging, termed sterile inflammation (indicating no detectable pathogens), is well described [15]. Inflammaging and inappropriate immune activation contribute to the pathogenesis of many age-related diseases, including diabetes, atherosclerosis, and AD [16]. Sporadic AD development has been linked to enriched risk genes that are present in aged microglia, the primary resident immune cell of the brain. This implicates brain innate immunity in neurodegeneration [17]. This concept is further supported by the presence of microglia with a senescent (dystrophic) phenotype in post-mortem AD brains [18]. The adaptive immune system and clonally expanded senescent T-cells also contribute to AD pathogenesis [19]. The risk of ischemic stroke and vascular dementia is increased with aging, in part due to increased reactive oxygen species (ROS) and enhanced coagulation induced by inflammation [20]. However, the mechanisms that drive age-related chronic inflammation in the brain and cerebral vasculature are not fully understood.

Both immunosenescence and inflammaging alter the microenvironment of the central nervous system (CNS), primarily by actions on microglia. Normally microglia are in a homeostatic state maintained by cellular signals and interactions with ligands that inhibit microglial activation. Ligands such as CD200, CXCL1, and CD47 are expressed by neurons and bind to corresponding receptors on microglia [21]. As neurons are damaged with age or vascular insults, these inhibitory ligand-receptor interactions with microglia are disrupted [21]. In addition, misfolded proteins, such as amyloid-beta (Aβ), accumulate during normal aging and lead to an increase in the levels of microglial pro-inflammatory cytokines [21–23]. With age, there is also an increase in the expression of specific cytokines, such as transforming growth factor-β (TGFβ). Chronic exposure of microglia to TGFβ impairs their capacity to secrete anti-inflammatory cytokines [24, 25] and leads to the downregulation of interferon regulatory factor-7, an important factor in switching microglia from a pro-inflammatory to an anti-inflammatory phenotype [25]. After an acute stroke, loss of blood brain barrier (BBB) integrity leads to a dramatic infiltration of peripheral immune cells into the brain, further contributing to neuroinflammation. The composition of these infiltrating cells in animal models differs between young and aged brains, as does their inflammatory potential [26].

## Cerebrovascular aging and immunity

**Fig. 1:**
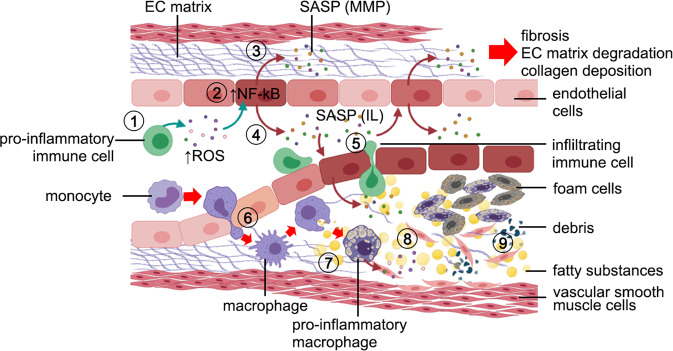
Age-related changes in the vasculature. [1] Senescent immune cells secrete reactive oxygen species (ROS) that [2] activates the NF-kB pathway in cerebral endothelial cells (CECs). Then, CECs adopt a senescent-associated secretory phenotype (SASP) and [3] secrete MMP that degrade the extracellular matrix. Other SASP components secreted by senescent CECs can also promote fibrosis and collagen deposition. [4] Senescent CECs secrete pro-inflammatory substances (IL-1, IL-6, IL-8) into the vasculature lumen that impair tight junctions between CEC, and [5] facilitate the infiltration of immune cells and monocytes through the CEC layer. [6] Infiltrating monocytes reach the internal elastic lamina and change their phenotype to macrophages, [7] which phagocytize oxidized lipoproteins. In the internal elastic lamina, [8] reactive macrophages and infiltrating immune cells secrete pro-inflammatory cytokines that exacerbate inflammatory responses, and [9] contribute to the deposition of cellular debris, fatty substances, migrated vascular smooth muscle cells, and lipid-laden macrophages (foam cells) that lead to the formation of atherosclerotic plaques. Figure made with Biorender.com.

A healthy cerebral vasculature is critical for brain function. Neuronal function and survival depend on the integrity of the brain’s blood vessels and their capacity to remove neurotoxic molecules from the interstitial fluid, including amyloid [27]. With aging, both venous and arterial tortuosity and vessel injury decrease cerebral blood flow (CBF), leading to the accumulation of beta-amyloid, hypoperfusion, and dysregulated exchange of nutrients [28]. The etiology of this tortuosity is unclear, but is due in part to adverse remodeling of the venular walls, impaired expression of angiogenic and growth factors, increased endothelial cell senescence, and dysregulation of matrix metalloproteinases (MMPs) [29] (See Fig. 1).

Age-related increases in arterial stiffness, chronic exposure to cell stress, endothelial senescence, and enhanced inflammatory processes are linked to the development of atherosclerosis [30]. The formation of atherosclerotic plaques is depicted in Fig. 1, led by macrophage infiltration into the arterial intima to phagocytose oxidized low-density lipoprotein [31]. These macrophages become active after ingesting lipids and secrete pro-inflammatory cytokines, further exacerbating vascular inflammation and increasing the size and complexity of the atherosclerotic plaque [32] (Fig. 1). Age-related changes in the cerebral vasculature can enhance white matter injury, a common feature in vascular dementia [33]. These myelinated white matter tracts create long-range connectivity and are involved in axonal transport, neuroplasticity, and learning. Endothelial dysfunction and hypoperfusion lead to demyelination and BBB breakdown, which is further exacerbated by the enhanced oxidative and inflammatory milieu seen in the aging brain [33].

Cerebral endothelial cells (CEC) are critical components of the BBB and contribute to its integrity, which is essential to maintain the balance of nutrients, immune cells, and overall brain homeostasis [34]. CEC dysfunction leads to BBB impairment and reduced blood flow in the proximity of white matter injury [35, 36]. Enhanced BBB permeability precedes the onset of dementia [35]. Thus, a healthy vascular endothelium is essential to maintain low and selective permeability of the BBB [37]. CEC, like most cell types, experience cellular senescence and “programmed” cell-cycle arrest with aging [38]. Endothelial senescence contributes to oxidative stress, inflammation, microvascular rarefaction, arterial stiffness, vascular luminal narrowing, and subsequent reductions in CBF. Thus, CEC senescence contributes to BBB impairment, an early step in vascular dementia.

Endothelial cells develop a pro-inflammatory genetic profile as they age, a shift that is activated by senescence-inducing factors, including NF-κB [39]. Endothelial cells are exposed early to cytokines produced by circulating senescent immune cells [40], which propagates the SASP. Senescent CEC then develop transcriptome changes that lead to increased production and secretion of pro-inflammatory factors [41], resulting in further deleterious effects in the cerebral vasculature [42–44] (see Table 1).

**Table 1:** Changes to gene and protein expression in aged endothelial cells in human and murine studies.

Target	Status	Phenotypic changes	Functional changes	Reference
TNF-α	↑	ROS production	Inflammation, NADPH oxidase activation, apoptosis	[218–221]
TNF-β	↑	ROS production	apoptosis	[218]
IL-1β	↑	ROS production	Inflammation	[219, 222]
IL-6	↑	Senescence	Increase adhesion molecules	[219, 223]
IL-6Rα	↑	Senescence	Inflammation	[219]
IL-17	↑	Senescence	Inflammation	[219]
MMPs	↑	Tight junctional complexes are disrupted	Weakened BBB becomes permeable vasoconstriction	[224–227]
MCP-1/CCR2	↑	Vascular remodeling	Leukocyte infiltration	[227–229]
VEGF	↓	Vasoconstriction	Reduced activation of eNOS	[230]
NADPH Oxidase	↑	Arterial remodeling	Activates MMPs, oxidative stress	[231]
Calpain-1	↑	Clot formation	ANGII/MMP signaling	[229, 232]
Local Ang II	↑	Arterial remodeling	Activates MMP-2, TGFβ	[233]
MFG-E8	↑	Increased concentrations in aortic wall	Activates MMP-2, TGF-β, collagen production	[224, 225, 227]
Nitric oxide Bio-availability	↓	Impaired cellular interactions	Decrease anti-fibrinolytic activity, apoptosis	[229, 234]
TGF- β 1	↑	Vascular remodeling	Increased collagen, fibrosis	[235, 236]
SIRT1	↓	Vascular Senescence	Reduced activation of eNOS and suppression of ANG II	[237] [238, 239] [240]

Reduced brain capillary density and impaired endothelial-dependent functions, including angiogenesis (critical for maintaining and modifying microvascular networks), is seen with aging. Impairments in endothelial-mediated vasodilation, and neurovascular coupling [45] also occur and further deteriorate the architecture of the cerebral vasculature [46–49] leading to subsequent cognitive impairment [50]. One increasingly common subtype of vascular dementia is cerebral amyloid angiopathy (CAA). CAA develops due to deposition of amyloid in the media and adventitia of small arteries and capillaries of the leptomeninges and the cerebral cortex. It is a leading cause of lobar intracerebral hemorrhage and cognitive impairment in the elderly. Although the hallmark of both CAA and AD is amyloid pathology, these diseases are clinically distinct. Less than 50% of CAA cases meet the pathologic criteria for AD and over 75% of patients with AD have only mild or no CAA [5, 51]. In CAA, vascular amyloid deposits cause inflammation, hemorrhage, and degradation of vascular smooth muscle cells and pericytes [52, 53]. Age is the most important risk factor for CAA [54]. These hemorrhages demonstrate the clear link between vascular and amyloid pathology [55–57].

Aged CEC are also directly implicated in the increased infiltration of peripheral immune cells, a hallmark of brain aging. CEC produce pro-thrombotic mediators and cellular adhesion molecules (i.e., intercellular adhesion molecule-1 and plasminogen activator inhibitor-1) in patients with vascular dementia. This increase in adhesion molecules amplifies the ability of immune cells to enter the CNS in response to vascular injury (Table 1). A subpopulation of angiogenic endothelial cells is induced in the brain of patients with AD [58]. These cells exhibit increased expression of angiogenic growth factors, their receptors (i.e., EGFL7, FLT1, and VWF), and antigen-presentation machinery (i.e., B2M and HLA-E). The contribution of endothelial cells to angiogenesis and immune responses in AD and other age-associated vascular diseases is increasingly evident. Importantly, some of the pathological changes seen in aged CECs are reversible and thus may be potential targets for therapeutic intervention [59]. Cerebrovascular aging connects the simultaneous deterioration of CEC function, negative alterations to cerebrovascular structure, impaired cerebral blood flow, impaired clearance of debris, and increased amyloid deposition that all contribute to cognitive impairment [60, 61].

## Microglial aging

**Fig. 2:**
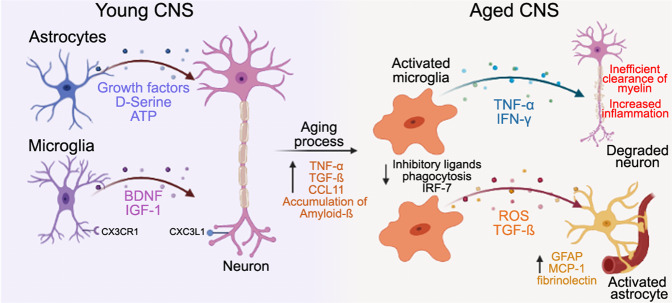
Astrocytes and microglia in a young CNS release growth factors and cellular signals to maintain homeostasis and control neurogenesis of neurons. As individuals age, cellular and molecular changes in the brain environment are initiated by an increase of pro-inflammatory cytokines and an accumulation of proteins, such as amyloid. Next, microglia and the innate immune response are activated, activating astrocytes and leading to neuronal damage. Activation of microglia and astrocytes disrupts the BBB and contributes to a heightened immune response and worse cognitive outcomes in elderly patients with cerebrovascular injury. Neurological signals: ATP: adenosine triphosphate, BDNF: brain-derived neurotrophic factor, CX3CR1 or CX3CL1: fractalkine receptor and ligand IGF-1: Insulin-like growth factor, Aβ: amyloid-beta, IRF-7: Interferon regulatory factor 7, INF-γ: Interferon gamma, GFAP: Glial Fibrillary acidic protein, MCP-1: Macrophage chemoattractant protein, ROS: Reactive oxygen species, TGFβ, transforming growth factor-β, TNFα: tumor necrosis factor-alpha. Figure made with Biorender.com.

The CNS has historically been considered to be an “immune privileged” site, but this concept is changing. Although microglia are the predominant innate immune cell in the CNS, there is robust communication between the brain and the peripheral immune system through a variety of different avenues, including through the CSF and lymphatics [62]. Microglia maintain a dynamic state of immune readiness by constantly scanning the brain environment for perturbations [63]. Microglia maintain brain homeostasis by interacting with signaling molecules secreted by healthy neurons [64, 65]. Young microglia and astrocytes promote angiogenesis, remodel the extracellular matrix, and suppress destructive immunity (see Fig. 2). Microglia play critical roles in both the developing and adult CNS; they react rapidly to danger signals, changing their morphology and adopting an activated state that can trigger the secretion of beneficial anti-inflammatory cytokines, such as IL-4 and IL-10 [66–68]. However, the microglia transcriptome changes with aging, leading to enhanced inflammation, impaired phagocytosis, and profound morphological changes that reduce immune surveillance [69].

A phenotypic hallmark of aging, and in numerous pathological conditions, is the emergence of “dark microglia” [70]. These microglia are found in close proximity to the vasculature and amyloid plaques in the brains of AD patients. They contain condensed and remodeled nuclear chromatin, an electron-dense cytoplasm, and exhibit features seen with oxidative and metabolic stress. However, microglial heterogeneity expands beyond dark microglia, and novel subtypes are emerging from transcriptomic data. For example, a microglial signature, enriched in AD susceptibility genes, has been found with human aging [71, 72], and a “disease-associated microglial” (DAM) profile has been described in neurodegenerative conditions in mouse models [73]. DAM cells cluster around amyloid plaques establishing a protective barrier, they are ApoE-Trem2 dependent and upregulate genes involved in lysosomal, phagocytic, and lipid metabolic pathways [73, 74]. TREM2 variants are associated with increased risk for sporadic AD supporting the involvement of microglial dysfunction in disease development [75]. In addition, supporting a specific role of dysfunctional and pro-inflammatory microglia, a novel lipid-droplet-accumulating microglial with defective phagocytosis and pro-inflammatory profile was recently identified in the aging brain [76].

Microglial dysfunction associated with aging contributes to cellular senescence and negatively affects the response to injury. These age-associated changes result in maladaptive immune responses, chronic inflammation, and poorer outcomes after CNS injury [77]. Aging skews the transcriptomic profile of microglia toward a chronic inflammatory state, upregulating genes involved in cytokine production, host defense, and cell adhesion [78–80]. Aged microglia have elevated levels of TNFα, IL-1β, and IL-6 (see Fig. 2) [81, 82]. In parallel, the loss of anti-inflammatory cytokines such as IL-10 suggests they have a reduced ability to restrain and control pro-inflammatory microglial pathways with aging (Fig. 2). Upregulation and transcription of genes involved in cell-cell interactions, immune cell chemotaxis, immune-inflammatory responses, and tissue remodeling/repair are reduced in microglia from aged compared to young mice. This is also seen in humans, aged human microglia have distinct upregulation of pathways associated with DNA damage, telomere maintenance, and phagocytosis [79, 83]. These shifts in microglial gene expression may be driven, in part, by IL-10 and IL-4 secreted by B-cells [84]. This suggests that aged subjects have difficulty mounting a controlled inflammatory response to injury, leading to detrimental effects on tissue repair.

## Peripheral immune cells contribute to brain immunosenescence

An increase in the number of resident immune cells is seen in the aged brain, both in animal models and in humans, including antigen-presenting cells (e.g., dendritic cells, T-cells, and B-cells) [85]. Under normal physiological conditions, the movement of peripheral immune cells is tightly regulated by the BBB. The delicate balance of cells and substances allowed to move between the cerebral blood vessels and the CNS is maintained by CEC and microvessels [86]. The transport of immune cells is regulated by a multi-step process involving interactions between adhesion and signaling molecules in endothelial and immune cells. Immune cells become tethered and attach to the blood vessels, allowing for the recognition of cytokines and specific carbohydrate ligands that then polarize immune cells. This identification process is necessary to strictly regulate which immune cells can enter the CNS [34]. Once an immune cell has been identified and polarized, it can cross the endothelial basement membrane. An increase in clonal and antigen-experienced T-cells with an effector memory phenotype is seen in the blood and cerebrospinal fluid of AD patients, implicating an adaptive immune response. These alterations are also seen in patients with other age-related neurodegenerative diseases [19]. What drives the increasing numbers of diverse immune cells to enter the CNS of aged individuals is unclear but likely involves age-related changes in the brain’s vasculature that facilitate their entry.

## Peripheral immune cell aging

The innate immune system comprises multiple cell types that rapidly recognize and react to conserved pathogen-associated molecular patterns and danger-associated molecular patterns (DAMPs) in a nonspecific manner [87, 88]. When the cerebral vasculature is damaged, peripheral immune cells are recruited to the site of injury via DAMPs and play a role in the inflammatory response and recovery of the CNS. Profound age-associated changes occur in many innate immune cell lineages, including neutrophils, dendritic cells, natural killer cells, and resident glial cells, which exacerbate CNS injury (Table 2).

**Table 2:** Inflammatory signal expression of aged immune cells and functional changes that arise from altered cytokine/chemokine production.

Cell Type	Total cell count	Inflammatory signal expression	ROS production	Phagocytosis	Overall function	Reference
Neutrophils	=	↑ G-CSF, IL-10, IL-4, IL-6, INFγ, TNFα	=	↑	↑ migration to inflammatory sites	[241]
Dendritic cells	↑	↑IL-6, TNFα	↑	↓	↓TLR expression	[242] [85]
Natural killer cells	↑	↑ INFγ	↓	N/A	↓cytotoxicity and chemotaxis	[243]
T-cells	Naïve: ↓ Memory: ↑	↑IL-6, TNFα ↓ IL-2	↑	N/A	↓ TCR repertoire	[244] [245] [246]
B-cells	Naïve: ↓ Memory: ↑	↑ IL-10, IL-4, TNFα	N/A	N/A	↓ clonal expansion	[247]
Microglia	↑ activated phenotype and dystrophic cells	↑ CCL11, TGFβ, IL-1β, IL-6, TNFα	↑	↓	↑ TLRs, MHCII	[248] [249]

## Aging in myeloid lineage cells

### Macrophages

Senescent hematopoietic cells secrete monocyte chemotactic protein (MCP)−1, which contributes to macrophage tissue infiltration [89]. The levels of infiltrated macrophages and pro-inflammatory cytokines are greater in the brains of 12-month-old mice (equivalent to a 40–45-year-old human) than in 6-month-old mice [90]. Once in the nervous tissue, macrophages activate either a pro-inflammatory (enriched with CD11c and the chemokine receptor CCR2) or an anti-inflammatory phenotype (enriched with CD163). Investigators have also used senescence-accelerated mouse (SAM) models (SAMP1, SAMP6, SAMP8, and SAMP10) and senescence-resistant mice (SAMR) to identify mechanisms of aging. The lifespan of SAMP strains is shorter than wild-type mice [91], and they exhibit accelerated senescence-associated phenotypes that copy those observed in age-related diseases in humans. For example, SAMP1 mice mimic amyloidosis, SAMP6 mice show symptoms of osteoporosis, and SAMP8 mice show age-dependent deficits in learning and memory. SAMP-1 mice have increased expression of macrophage markers (F4/80) in the brain. Expression of other macrophage markers (CD11c, relative to CD163) and CCR2 levels are also higher in the brains of SAMP1 mice compared to control mice, suggesting that macrophages reach the brain from the bloodstream and become pro-inflammatory with senescence [92]. This phenotype is accompanied by increased expression of MCP-1 in SAMP1 mice [92], mirroring what occurs in natural aging models.

Macrophages, in conjunction with activated microglia, migrate into the brain after a cerebrovascular insult to phagocytose debris from apoptotic neurons. This event occurs in the cerebral cortices of young mice even after a micro-infarct [93]. This controlled macrophage infiltration is a beneficial inflammatory response, enhancing debris clearance and preventing further brain injury. However, this may not be the case in an aged brain, as macrophages impair synaptic plasticity in the hippocampus of aged mice [90, 94]. Bone marrow macrophages incubated with soluble brain extract from aged mice had increased expression of MHCII and CD40 compared to macrophages incubated with extracts from the young brain. When these macrophages were primed with the inflammatory cytokines seen in aged brain and applied to hippocampal slices, long-term potentiation was inhibited [90]. This suggests that infiltrating macrophages can respond to the local inflammatory milieu seen in the aged brain. The mechanisms driving macrophages responses and their influence on synaptic plasticity are unknown. It is hypothesized that the systemic increase in inflammatory cytokines found in aged individuals leads to phenotypic changes in macrophages, which can damage neurons, impairing synaptic plasticity. Additionally, aged macrophages produce prostaglandin E2, which can inhibit T-cell growth and proliferation, and CCR6 [95], leading to additional recruitment of macrophages. Thus, the role of macrophages depends on a delicate balance of cytokine levels, which is disrupted with brain aging.

### Neutrophils

Neutrophils have multiple host-defense functions, including enzyme secretion, phagocytosis, cytokine production, and generation of ROS and neutrophil extracellular traps [96]. Aging is associated with deficits in neutrophil recruitment, including decreased accuracy of neutrophil chemotaxis and migration toward inflammatory stimuli [97]. Aged neutrophils exhibit dysfunction in debris clearance and production of enzymes needed for vascular remodeling [98]. Similar to macrophages and microglia, neutrophils’ ability to clear cellular debris is critical to their anti-inflammatory, immune response. Altered phagocytosis, neutrophil extracellular traps release, and enhanced ROS generation in neutrophils from aged hosts have the potential to impair the response to infection or sterile injury and worsen chronic inflammation within healthy tissue [99]. Thus, with aging, neutrophils become less resilient and less functionally efficient.

### Dendritic cells

Dendritic cells are integral for antigen presentation and preserve the equilibrium between immune tolerance and aberrant immune responses [100]. Age has profound effects on dendritic cells, both at baseline and with stimulation [101, 102]. Aged dendritic cells have reduced ability to induce proliferation of CD4+ and CD8+ T-cells and to stimulate these cells to secrete interferon (IFN)-γ [103, 104]. Aged dendritic cells have impaired antigen uptake and phagocytosis of apoptotic cells, which prolongs self-antigen exposure and promotes chronic auto-inflammation in elderly hosts [101]. In addition, dendritic cells play an important role in neurodegenerative disorders, cerebrovascular disease, and cancer [105–108], which are pathologies associated with aging. In the aging murine brain, major histocompatibility complex-II expression increases in peripherally sourced myeloid antigen-presenting cells, including dendritic cells. These cells continue to accumulate in the brain with advancing age [85]. This increase in brain dendritic cells correlates with the emergence of age-associated behavioral deficits, but further studies will be needed to directly assess the causal role of dendritic cells in brain aging.

### Natural killer cells

Natural killer cells prevent viral infections and tumor growth. However, their numbers are significantly increased in the postmortem brain tissue of aged humans [109] and aged mice [110]. Jin et al. recently proposed a mechanism that links natural killer cell activation with cytotoxicity and cognitive dysfunction in the hippocampi of aged mice. These activated natural killer cells are identified by perforin and granzyme B (cytotoxicity mediators) and CD96 and NKG2D (activation markers) [110]. Neuroblasts become senescent in the aged brain and secrete IL-27, which promotes natural killer cell proliferation and activation. The temporal depletion or reduction of these cells in old mice enhanced the numbers of neuronal precursors and reduced apoptotic neuroblasts in the hippocampus and led to improved cognitive function and enhanced synaptic plasticity [110]. These findings could help to target natural killer cells in neurodegenerative disorders.

## Aging in the lymphoid lineage

The adaptive immune system consists of lymphocytes, including T- and B-cells, which are antigen-specific and create long-lived immune memory. These cells have a much larger role in age-related CNS dysfunction than previously recognized.

### T-cells

As aging progresses, a decrease in naive T-cells leads to a shrinking of the T-cell receptor (TCR) repertoire, which may be a consequence of thymic involution and chronic antigenic stimulation [111, 112]. The TCR repertoire is necessary for the response to infection, and the loss of TCR diversity in elderly patients may make them more vulnerable to infections [113]. Aging is also associated with an accumulation of expanded clones of memory and effector T-cells, believed to result from lifelong exposure to continuous oxidative stress and antigens [114–117]. One consequence of aging is the decreased ability of aged naive CD4^+^ T-cells to interact with antigen-presenting cells and respond to antigens in general. Thus, CD4^+^ T-cells from aged mice do not expand, produce cytokines, or differentiate as effectively as in young mice [118]. In addition, mouse and human regulatory T-cells, which suppress the immune response, become more numerous and increase their function with aging [119–122]. This can cause a dysregulation in the immune signals that would normally aid in beneficial immune cell interactions and controlled inflammatory responses.

### B-cells

The production of naive B-cells in the bone marrow declines with aging, potentially due to age-associated inhibition of genes required for B-cell precursor maturation [123]. As a result, clonal expansion, cytokine production, and antibody production in response to new challenges are impaired, leaving elderly hosts at greater risk of infection, cancer, and other chronic diseases [123].

Similar to T-cell immunosenescence, aging leads to fewer novel B-cells and more aged antigen-specific B-cells [124, 125]. Age-associated B-cells are found in both mice and humans [126, 127]. In animal models of AD, levels of activated B-cells are increased in the circulation, and enhanced infiltration of B-cells into the CNS results in immunoglobulin deposits around Aβ plaques. AD progression requires B-cells, as the loss of these cells alone is sufficient to reduce Aβ plaque burden and activity of disease-associated microglia. B-cell depletion reverses behavioral and memory deficits, restores TGFβ^+^-microglia, and slows AD progression in mice [128]. The role of B-cells in stroke will be discussed below.

In summary, age-related alterations occur in all major peripheral immune cell subsets (Table 2). These alterations contribute to immune dysregulation/inflammaging and a skewing of the immune response towards increased basal chronic inflammation, impairing host defenses and contributing to the pathogenesis of both acute and chronic inflammatory diseases.

## Age-related changes in stroke-induced inflammation

### Ischemic stroke pathology

After ischemic heart disease, stroke ranks as the second leading cause of death worldwide. Acute ischemic stroke, accounting for ~87% of all strokes, is caused by the loss of cerebral blood flow [129]. Secondary damage pathways intensify tissue injury for days or weeks after the initial event [130]. This ongoing sterile inflammation contributes to secondary damage after ischemic stroke [131]. Fortunately, most ischemic stroke patients survive their initial injury. However, poor functional outcome in stroke survivors is a major determinant of overall disease burden. There are ~6 million stroke survivors living in the United States, and this number is projected to increase to 10 million by 2030 as the aging population expands [129]. The economic cost of care increases with age as elderly stroke survivors (≥65 years) are more likely to have severe deficits, other co-morbid illnesses, and require greater care [132, 133].

### The inflammatory response to stroke

**Fig. 3:**
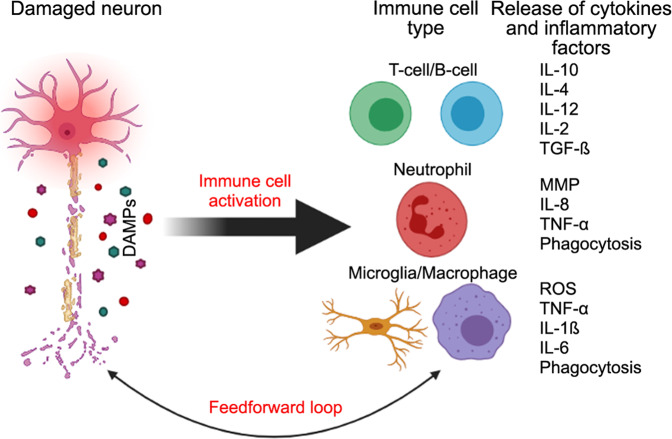
Cellular and molecular changes in the brain are initiated by primary brain injury. In response to injury, damage-associated molecular patterns (DAMPs) are released, and an innate immune response characterized by glial activation and infiltration of blood-borne immune cells into the brain occurs. The activation and infiltration of peripheral immune cells lead to secondary brain injury, further destroying brain tissue and poor recovery. Figure made with Biorender.com.

Inflammation protects the host from pathogens, clears dead cells, and facilitates tissue repair after injury. The body produces an inflammatory response to infection or tissue damage; once the agent is removed, inflammation is resolved, allowing the tissue to return to homeostasis [134]. Brain ischemia quickly causes failure of ion pumps, over-accumulation of intracellular sodium and calcium, loss of membrane integrity, and necrotic cell death [135]. DAMPs, also known as alarmins, are released by necrotic and dying neurons that stimulate the inflammatory response [136] (see Fig. 3). DAMPs include a variety of molecules ranging from extracellular proteins, intracellular proteins (i.e., high-mobility group box 1 proteins and heat shock proteins) to plasma proteins like fibrinogen. Once in the bloodstream, DAMPs bind to pattern recognition receptors of peripheral immune cells and initiate the post-stroke inflammatory response, including cytokine release as these cells are recruited into the brain [137]. For example, monocytes and neutrophils are activated outside of the brain and are recruited to the site of ischemic injury to assist in repair and recovery [138] (Fig. 3). However, how these cells are primed in the periphery matters. Once they leave the bone marrow niche, they are exposed to a plethora of cytokines in the aged blood that contributes to their pathogenicity [139].

One of the first studies to directly examine the immune response to stroke in aged animals compared young-adult (5–6 months), middle-aged (14–15 months), and aged (20–22 months) C57BL/6 male and female mice using a transient middle cerebral artery occlusion model [140]. Acute functional outcomes were worse with aging, with a concomitant increase in neutrophils, inflammatory macrophages, dendritic cells, and activated microglia in the aged brain, which may contribute to the greater behavioral deficits and higher mortality seen in aged mice [26, 140].

In the aged brain, chronic immune activation and inflammation contribute to further neurodegeneration and tissue loss. The inflammatory response impairs neurogenesis and contributes to poor functional recovery [141]. Elevated innate immune cell responses within 48 h of a stroke are associated with poor cognitive recovery [138]. The removal of the largest pool of peripheral immune cells via splenectomy reduces injury in aged mice after stroke and decreases stroke induced inflammation [138]. Therefore, reducing the amount of aged peripheral immune cells recruited to the ischemic brain can improve cognitive recovery post-stroke [142].

It is increasingly clear that it is not just the amount of infiltrating immune cells that differs in the young versus the aged brain; the temporal pattern and cell type that enters the brain also contributes to stroke outcome [139]. There are marked differences in the composition of circulating and infiltrating leukocytes recruited to the ischemic brain of aging mice compared to young mice. Aged animals exhibited enhanced levels of neutrophils in the blood and had more neutrophil invasion into the brain. These infiltrated neutrophils had reduced ability to phagocytize pathogens and debris and expressed high levels of extracellular matrix-degrading enzymes (i.e., MMP-9) and markers of oxidative stress in aged animals. Aged mice had more pronounced hemorrhagic transformation compared with young mice relative to infarct size, which may reflect their increased MMP expression. In humans, higher numbers of myeloperoxidase-positive neutrophils were found in postmortem brain samples of old (>71 years) ischemic stroke patients compared with age-matched controls. Neutrophils were found in the human brain parenchyma, and a significant proportion of these were MMP-9-positive, and found in areas of hemorrhage and hyperemia. These age-related changes in the myeloid response to stroke suggests that the bone marrow response to stroke is also altered with age. To directly evaluate this, heterochronic bone marrow chimeras were generated to determine the contribution of peripheral immune senescence to age- and stroke-induced inflammation [26]. Aged host mice that received young bone marrow had attenuation of age-related reductions in bFGF and VEGF. They also had improved locomotor activity and gait dynamics compared to isochronic controls (old mice reconstituted with old bone marrow), even in the absence of ischemic injury. Microglia in young heterochronic mice (that received old bone marrow) developed a senescent-like phenotype. Cohorts of animals were subjected to transient middle cerebral artery occlusion. Aged mice that received young bone marrow had improvements in post-stroke behavioral deficits and had fewer brain-infiltrating neutrophils compared with isochronic controls. Young mice reconstituted with aged bone marrow had higher rates of hemorrhagic transformation, increased mortality, and worse behavioral outcomes. This implies that an aged peripheral immune system negatively affects the immunological response to stroke, even when the animal was young. More importantly, from a translational perspective, these detrimental effects were reversed by manipulation of the peripheral immune cells in the bone marrow [26].

Similar studies have also targeted peripheral inflammation as an approach to reduce ischemic injury. A major source of antigens and immune cells is the gut. Aged animals given a fecal transfer of a “young” microbiome also had improved stroke outcomes compared to aged animals reconstituted with an aged biome [139]. This protection was related to an enhancement in the integrity of the gut barrier and attenuation of the inflammatory response in both the gut and brain. Young biome augmented the frequency of intestinal Treg cells and reduced inflammatory brain IL-17^+^ γδ T cells levels in aged hosts. Beneficial effects of youthful biome also extend to cognitive function. Mice raised in germ free (GF) conditions that were transplanted with young donor microbiome had improved cognitive performance compared to GF mice reconstituted with aged biome [140]. This suggests that there is the potential to reverse “inflammaging” via manipulation of peripheral tissues, an area of active investigation in both vascular and neurodegenerative diseases [143, 144].

### Glial contributions to ischemic stroke in aging models

An enhanced glial response and higher pro-inflammatory cytokine production have been seen in aged animals after stroke [145, 146]. Surprisingly, aged mice (16 months) have smaller infarct volumes and less edema than younger male mice (9–12 weeks) [147]. Similar results were found in rat models of stroke with greater histological damage in young (3 months) compared to old (24–26 months) male rats [148]. Surprisingly, aged animals, which exhibit small infarcts, manifested higher mortality and more severe behavioral deficits than young mice. A more rapid development of the infarct, enhanced glial scarring, and a delayed suboptimal functional recovery were seen after stroke in aged animals [146, 149].

**Fig. 4:**
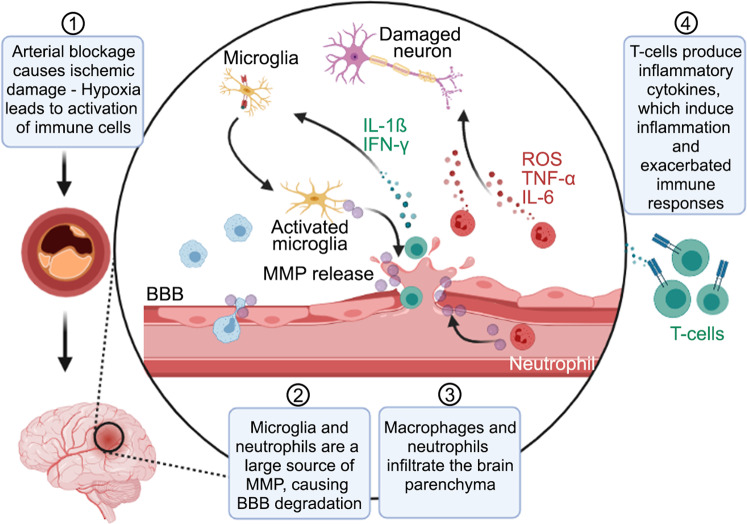
After a brain insult, including ischemic stroke, activated microglia trigger an inflammatory response and eliminate debris from apoptotic cells. After ischemic stroke, immune cells activate [1], and microglia secrete MMPs [2] that disrupt the integrity of the BBB and facilitates the invasion of macrophages and neutrophils into the brain parenchyma [3]. However, in the aged brain, this pro-inflammatory response is extended and contributes to the participation of T-cells that magnify the immune response [4]. Depletion of microglia prior to stroke exacerbated injury. One potential strategy to mitigate inflammation after brain injury is to deplete pathological microglia or enhance their capacity for repair. Figure made with Biorender.com.

In response to stroke, microglia are one of the first responders, quickly developing an activated phenotype, generating ROS, phagocytizing, and producing pro-inflammatory cytokines and proteases [26] (Fig. 4). These activated microglia phagocytose dying cells and debris and are necessary for later repair. After stroke, the percentage of phagocytosing microglia increased at 24 h and peaks by 72 h [150]. Due to this extended period of activation, dysregulation of this response contributes to poorer stroke outcome and secondary damage. Colony-stimulating factor 1 receptor (CSF1R) signaling is required for microglial survival [151]. A highly specific CSF1R inhibitor (PLX5622) can be orally administered to deplete microglia. Young mice depleted of microglia had increased infarct size after stroke [152]. However, as microglia may be pathologically activated at baseline in the aged brain, we tested if depletion could lead to beneficial effects in aged models. Aged animals (18–19 months), depleted of microglia fed (PLX5622) for 3 weeks before an induced stroke, also had increased infarct damage and myeloid cell infiltration at 24 and 72 h after stroke [153]. Despite the dysregulated state of aged microglia, this experiment suggests that aged microglia have some beneficial effects during the acute phase of ischemic stroke. Similar findings have been reported in aged rats, where cognitive function and synaptic transmission benefit from the support of aged microglia, and removal of these cells was deleterious [154]. However, the chronic effects of microglia depletion and repopulation on cognition and stroke repair remain to be investigated (Fig. 4).

Astrocytes expressing IL-15 are one of the first cells that recruit microglia to the damaged area. This enhances their differentiation into a pro-inflammatory phenotype, worsening neurological outcomes after stroke in mice [155]. Specifically, chemokines, such as C-C motif chemokine ligand-2 (CCL2), increase pro-inflammatory functions of microglia and recruitment of peripheral macrophages to the injured brain [156]. Deficits in age related cognitive recovery after stroke can often be traced back to the chemical signals received and secreted by immune cells in the early phase of injury.

Glial cells also secrete MMPs, which maintain the integrity of the basement membrane and are essential for BBB maintenance (Fig. 4). MMPs degrade components of the extracellular matrix (collagen, laminin, fibronectin, and proteoglycans) [157]. MMPs are produced and secreted by CECs, neurons, glial cells, and peripheral immune cells, such as neutrophils [26]. With aging, the balance between MMPs and the extracellular matrix is impaired, leading to aberrant degradation of the extracellular matrix or enhanced collagen deposition or fibronectin [158]. Microglia are also a major source of MMPs after stroke, especially MMP-3 and MMP-9 [159, 160]. MMP-9 is linked to increased BBB disruption and can lead to poorer stroke outcomes in older patients due to increased peripheral immune cell activation [161] (see Fig. 4). Reduced disruption of the BBB is also important for regulating the influx of peripheral immune cells and hemorrhagic transformation [142, 162, 163]. Acute MMP inhibition reduces infarct size, brain edema, and recombinant tissue plasminogen activator–induced hemorrhage in animal models [161, 164]. Mice deficient in MMP-3 or MMP-9 have less ischemic injury than wild-type controls [165, 166]. Higher serum levels of MMP-9 in aged animals predict poor outcome and infiltrating neutrophils in aged mice produce higher levels of MMP-9 than those of young animals [167]. Extracellular MMP inducer (EMMPRIN or CD147) is a cell-surface glycoprotein that induces production of MMPs, including MMP-9. Patients with chronic inflammation have higher levels of CD147 [161, 168, 169]. Blocking CD147 with an antibody reduced brain hemoglobin and MMP-9 levels in mice 3 days after stroke and reduced infarct size and behavioral deficits. In stroke patients, high levels of serum CD147 24 h after stroke predicted poor functional outcome at 12 months. The levels of CD147 in the brain positively correlated with MMP-9 and secondary hemorrhage in post-mortem stroke patient samples [170].

While the immediate effects on BBB breakdown are apparent in stroke, chronic changes in BBB integrity also allow for increased entry of cells of the slower acting adaptive immune response. Increased numbers of CD8^+^ T-cells are found in the aged brain parenchyma, choroid plexus, and meninges in mice [171]. These cells have effector memory (CD44^+^, CD62L^−^), tissue-resident phenotypes, and expressed markers associated with TCR activation. The CD8 T-cell levels negatively correlate with pro-inflammatory function of microglia. However, after stroke or ex vivo stimulation, these cells dramatically increased their production of TNF, IFN-γ, and MCP-1/CCL2. This population of resident memory, immune-surveilling CD8 T-cells is a hallmark of CNS aging. They modify microglia homeostasis under normal conditions, but are primed to potentiate inflammation and leukocyte recruitment after ischemic injury [171]. CD8^+^ T-cells also inhibit neurite growth, further impairing stroke recovery [172].

Adoptive transfer of regulatory T-cells reduced inflammatory responses both intrinsic and extrinsic to the CNS [173]. Moreover, T regulatory cells provide neurovascular protection against stroke by inhibiting peripheral neutrophil-derived MMP-9 production, but these studies were only performed in young animals [174]. Clearly, the balance in the immune cell subtypes is critical to stroke outcome, but studies in aged animals are needed. CD4^+^ T-cells also have a differential response to stroke in the aged brain. CD4^+^ T-cells secrete IFN-γ, stimulating the release of C-X-C motif chemokine ligand (CXCL)−10 from multiple cell types [175]. CXCL10, in turn, stimulates CD4^+^ T-cells to secrete more IFN-γ and other pro-inflammatory cytokines. Aged mice had significantly higher levels of CXCL10 in the serum and post-stroke brain than young mice. Behavioral recovery after experimental stroke was improved in aged mice depleted of CD4^+^ T-cells [176]. CD4 depletion reduced levels of pro-inflammatory cytokines, such as IFN-γ, CXCL10, CCL2, and CXCL1 [176], and lower levels of CXCL10 were linked to improved cognitive recovery. As depicted in Fig. 4, aged T-cells can secrete inflammatory cytokines and influence the inflammatory response after stroke, leading to downstream activation of other immune cells that impact cognitive outcomes.

B-cells are also recruited to the ischemic brain by CXCL13 [177]. Young mice lacking B-cells have increased infarcts, functional deficits, and mortality [178]. Adoptive transfer of B-cells reduced infarct volumes. Young mice depleted of B-cells by a humanized antibody to CD20^+^ (rituximab) had delayed motor recovery, impaired spatial memory, and reduced stroke-induced hippocampal neurogenesis weeks after stroke. However, no studies have confirmed these findings in aged mice. In vitro studies show that B-cells exert a direct neuroprotective effect on neurons and preserve neuronal dendritic arborization after oxygen glucose deprivation [179]. Brain tissue from human stroke and dementia patients were compared for B-cell density and IgG immune reactivity. Data from these animal models, coupled with human data, found an association between self-reactive antibodies and cognitive decline. These data are consistent with previous studies that reported persistent immune cell infiltration, even decades after stroke [180]. Importantly, further studies are needed to clarify the contribution of B-cells to post-stroke injury and repair and how this is related to aging.

## Clinical evidence of differences in the immune response to stroke in aging

One of the first studies to examine the adaptive immune response to stroke was performed in 2004 [181]. Peripheral blood CD4^+^CD28^-^ cells were collected from patients (75±13.5 years, 50% female) within the first 48 h of ischemic stroke and analyzed by flow cytometry. Rising counts of circulating CD4^+^CD28^-^ cells were associated with an increased risk of stroke recurrence and death over the next year. Expansion of this T-cell subset was suggested as a contributory pathogenic mechanism of recurrent stroke and death after ischemic stroke [181]. Clinical studies of stroke patients (71.8±14.4 years, 36% female) found IL-17-secreting T-cells in the peripheral blood 30 days after stroke. These IL-17 levels were associated with poorer cognitive status in post-stroke patients [182, 183]. Additionally, perivascular CD4^+^ T-cells in acute stroke lesions from post-mortem human samples secrete IL-21, a mediator of inflammation [184, 185]. Patients (73.4±15.7 years, 40% female) with acute ischemic stroke had increased expression of toll-like receptor (TLR)−4 on peripheral blood monocytes. Increased TLR4 expression correlates with increased stroke severity [186]. TLR4 mediates the activation of innate responses in monocytes, such as NF-κB activity and TNF-α synthesis, and are associated with worse outcomes in stroke patients [187]. Aging alters the immunological response to stroke and, consequentially, the post-stroke recovery process.

## Inflammation in other age-related cerebrovascular diseases

### Inflammation in vascular contributions to cognitive impairment and dementia (VCID) with aging

VCID is a heterogeneous group of disorders characterized by cognitive deficits secondary to cerebrovascular pathology. Chronic cerebral hypoperfusion is important in the onset of VCID. After AD, VCID is the second most common cause of dementia and accounts for ~15% of all dementia cases when it occurs as a single dementia diagnosis. Many dementia patients have mixed dementia (i.e.,VCID and AD pathologies) [188]. The risk of dementia after a cardiovascular event varies by its severity and the incidence of VCID increases with age [189].

People over the age of 65 are at the highest risk for VCID [188]. A stroke patient has two-fold increased risk of developing dementia compared with an individual with no history of stroke. This risk is highest within the 6 months after stroke; however, an increased risk prevails for at least a decade, even after controlling for known dementia risk factors [190, 191]. Although there are no treatments for post-stroke dementia, recent evidence has improved our understanding of the mechanisms that contribute to cognitive decline. What molecular mechanisms govern high risk of dementia incidence in stroke survivors are not identified; however, growing evidence from clinical and pre-clinical studies suggests that there exists a connection between neuroinflammation and cognitive decline. Thus, chronic brain inflammation caused by defective elimination of harmful substances and exacerbated and extended immune response may contribute to post-stroke dementia. The role of immunosenescence and inflammaging in cerebral small vessel disease has been recently reviewed [192].

Endothelial dysfunction and subsequent BBB leakage are the most critical mechanisms leading to VCID. Post-mortem brain tissues from 80 to 90-year-old humans exhibited SASP phenotypes in their cerebral microvessels [193]. The presence of senescent CEC is associated with enhanced BBB permeability due to SASP [194]. Importantly, BBB leakage was observed near areas of white matter injury in VCID patients, and it has been implicated in mild cognitive impairment and neurodegeneration [35, 195, 196]. The hippocampus and the striatum of patients with neurodegenerative disorders show high levels of pro-inflammatory cytokines associated with senescence [197]. Accelerated-senescence mouse models also exhibit enhanced senescent CEC, early and severe BBB integrity loss, and cognitive dysfunction [198, 199]. Understanding the molecular mechanisms that drive CEC to senescence during aging will help to identify potential therapeutic targets for age-related cerebrovascular diseases and dementias.

**Fig. 5:**
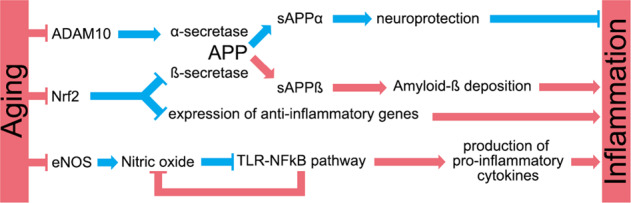
Aging has detrimental effects in the regulation of important anti-inflammatory regulators associated with the cerebral vasculature. The expression of ADAM metallopeptidase domain 10 (ADAM10), nuclear factor erythroid 2-related factor (Nrf2), and endothelial nitric oxidase synthase (eNOS) is downregulated with aging. ADAM10 cleavages amyloid-β precursor protein (APP) and forms soluble APPα, which opposite to soluble APPβ appears to be neuroprotective. Nrf2 negatively regulates the expression of β-secretase, which cleavages APP and form soluble APPβ. This contributes to the deposition of amyloid-β and promotes neuroinflammation. eNOS is synthesized by the cerebral endothelium and prevents oxidative stress. However, reduced levels of eNOS during aging enhances oxidative stress, which activates the TLR-NF-κB pathway axis and enhances the secretion of pro-inflammatory cytokines. In addition, the TLR-NF-κB pathway axis uncouples eNOS, creating a feedback loop that aggravates neuroinflammation. Blue indicates a beneficial effect for the cerebral vasculature and the brain, and red indicates a harmful effect.

Neuroinflammation has been linked to dysfunction of the cerebral endothelium. Similar to stroke, enhanced levels of infiltrating immune cells occur in patients with cerebral small vessel disease, which precedes VCID [200]. Senescent endothelium in aged VCID patients could lead to cerebrovascular inflammation. Endothelial nitric oxidase synthase, which produces the anti-inflammatory and anti-oxidative molecule nitric oxidase, is downregulated with aging [201]. Downregulation of nitric oxidase levels results in reduced CBF, vessel tone dysfunction, and enhanced oxidative stress, which activates the (TLR)-NF-κB pathway and enhances the secretion of pro- inflammatory cytokines [202]. TLR-NF-κB-induced oxidative stress can also uncouple endothelial nitric oxidase synthase and impair nitric oxidase production. Thus, endothelial cells enter in a vicious cycle of nitric oxidase downregulation, oxidative stress, and inflammation that eventually leads to cerebrovascular dysfunction and cognitive impairment (Fig. 5). Using antibodies against pro-inflammatory cytokines is a promising approach to prevent inflammation in the cerebral vasculature to mitigate phenotypes associated with vascular dementia. For example, targeting TNFα, which is upregulated with aging, restored endothelial nitric oxidase synthase levels, ameliorated motor and cognitive function, and reversed vascular endothelial dysfunction [203]. Chronic cerebral hypoperfusion also has detrimental effects on the brain. Experimental bilateral common carotid artery stenosis induces upregulation of MMP-1 and MMP-9, which degrade collagen I/III. This is associated with reduced cross-sectional area, wall thickness, and wall-to-lumen ratio in major arteries that supply the brain [204]. Aged CEC exhibit changes in gene expression that lead to inflammation and endothelial senescence. These transcriptional changes may be caused by downregulation of master genetic regulators during aging, such as nuclear factor erythroid 2-related factor (Nrf2).

The activity of the pro-survival and anti-oxidant Nrf2 declines in aged individuals across multiple species. The components of the Nrf2 pathway are downregulated in CECs and vascular smooth muscle cells in peripheral vessels [205] (see Fig. 5), as well as in the cerebrovasculature of aged non-human primates [206]. Nrf2 deficiency impairs neurovascular coupling, increases amyloid β precursor protein (APP) levels, increases neuroinflammation, and induces cognitive impairment [207]. Nrf2 depletion in aged mice also increased the expression of senescence-associated genes and microglia activation-related genes in the hippocampus [208]. Mouse models of VCID also demonstrate increased levels of Nrf2 but may be species specific [209]. Upregulation of Nrf2 in neurons may mitigate VCID [210]. However, whether targeting Nrf2 specifically in the cerebral vasculature can prevent the vascular-associated pathobiology seen in VCID has not been explored.

### Inflammation in CAA, a hallmark age-related vascular disease

CAA is caused by progressive Aβ deposition within the cortical and leptomeningeal arteries in the elderly, which causes intracerebral micro-bleeds, hemorrhages, inflammation, endothelial cell dysfunction, and death. The greatest risk factor for CAA is advancing age [54]. 20–40% of postmortem human samples show CAA pathology, and 80% of AD patients have Aβ deposition in their cerebral vasculature [211], suggesting that Aβ accumulation along cerebral blood vessels contributes to dementia. Aβ deposition has dramatic consequences in the cerebrovasculature that lead to a rarefaction of pial collateral vessels, the primary source of protection after ischemic insults in the brain [212]. CAA contributes to cerebral hypoperfusion, and hypoperfusion accelerates Aβ deposition in a positive feedback loop that aggravates CAA pathology [213]. Amyloid-beta deposition in the media and adventitia layers leads to degeneration of vascular smooth muscle cells and pericytes and vascular fragility, inflammation, and cerebral micro-bleeds [53]. These small hemorrhages occur in 17–46% of patients with cognitive impairment and demonstrate a clear link between vascular and amyloid pathology [55–57].

Aging is a risk factor for Aβ accumulation in the brain vasculature, as it downregulates key players in the metabolism of APP. The protease ADAM metallopeptidase domain 10 (ADAM10) cleavages APP to soluble APPα [214], which is neuroprotective. ADAM10 is downregulated in senescent cells, and reduced levels of this metallopeptidase are associated with neuroinflammation and immune activation [215] (Fig. 5). Symptomatic CAA mice have more activated resident and infiltrating myeloid cells than pre-symptomatic CAA mice [216]. In a murine model of CAA, anti-inflammatory polyunsaturated fatty acid metabolites prevented Aβ deposition along cerebral blood vessels and reduced neuroinflammation [217]. Thus, ADAM10 appears to be a potential target to mitigate Aβ accumulation and prevent CCA.

## Conclusion and future directions

**Fig. 6:**
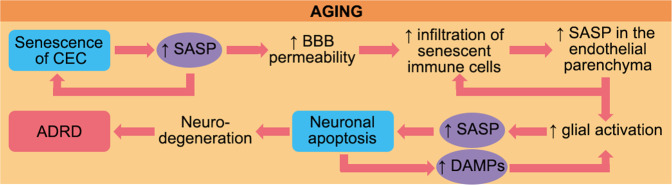
Aging is the main risk factor for Alzheimer’s disease and related dementias (ADRD). Aged individuals show an enhanced number of senescent cerebral endothelial cells (CECs) in their brains, which acquire a senescence-associated secretory phenotype (SASP) with detrimental consequences for tight junctions and the blood brain barrier (BBB) integrity. Thus, circulating senescent immune cells are more likely to penetrate between CECs and infiltrate into the endothelial parenchyma, where activated macrophages secrete pro-inflammatory cytokine. Glial cells also secrete molecules that contribute to the inflammation of the CNS and promote neuronal apoptosis. Further, danger-associated molecular patterns (DAMPs) derived from apoptotic neuron debris contribute to glial activation that sustains the CNS inflammation. Altogether, these events lead to neurodegeneration and contribute to ADRD and VCID. Note that SASP are “hot points” that exacerbate inflammation in the aged brain. Targeting the secretion of pro-inflammatory molecules from senescent cells and blocking the harmful feedback loops on CECs and neurons may prevent BBB disruption and neurodegeneration and thus counteract age related brain changes.

During aging, the immune system’s capability to maintain an effective response is dramatically decreased, causing chronic, uncontrolled inflammation that can trigger or accelerate age-related brain disorders. The events and subsequent feedforward loops caused by aging are summarized in Fig. 6. Aged individuals have senescent cells in their cerebral vasculature that may trigger peripheral immune cell infiltration. Circulating immune cells also undergo age-related changes that disrupt tight junctions between endothelial cells and impair BBB integrity. This allows for immune cell entry into the brain, including myeloid cells that secrete pro-inflammatory cytokines and metalloproteinases. Secreted cytokines quickly reach microglia and astrocytes, promoting a further inflammatory response. Aging exacerbates the response of activated astrocytes and microglia, leading to sustained recruitment of peripheral cells involved in both innate and adaptive immunity. The altered immune response to stroke worsens neurological outcomes and may majorly contribute to the disparate outcomes between young and aged patients. Levels of pro-inflammatory immune cells are positively correlated with worse outcomes in stroke patients, days and weeks after the insult. Similar observations have also been made in animal models.

In conclusion, many cell types and cellular components are involved in immunosenescence and inflammaging. There are a multitude of factors and pathways that are disrupted with aging, including the loss of anti-inflammatory mechanisms. The reduced capacity of the aged immune system to downregulate its response leads to an increase of pro-inflammatory cells and diminishing anti-inflammatory mediated repair of injury. Therefore, the biological age of the immune system may be a better predictor of the immunological response to cerebrovascular injury than chronological age. Novel approaches to reduce age-related brain inflammation via manipulation of peripheral immunity may hold great promise for the treatment of vascular and neurodegenerative diseases.
